# Role of toll-like receptors in post-COVID-19 associated neurodegenerative disorders?

**DOI:** 10.3389/fmed.2025.1458281

**Published:** 2025-03-26

**Authors:** Senthil Kumaran Satyanarayanan, Tsz Fung Yip, Zixu Han, Huachen Zhu, Dajiang Qin, Suki Man Yan Lee

**Affiliations:** ^1^Centre for Regenerative Medicine and Health, Hong Kong Institute of Science & Innovation, Chinese Academy of Sciences, Hong Kong Science Park, Hong Kong, Hong Kong SAR, China; ^2^School of Public Health, Li Ka Shing Faculty of Medicine, The University of Hong Kong, Hong Kong, Hong Kong SAR, China; ^3^Key Laboratory of Biological Targeting Diagnosis, Therapy and Rehabilitation of Guangdong Higher Education Institutes, The Fifth Affiliated Hospital of Guangzhou Medical University, Guangzhou, China; ^4^Bioland Laboratory, Guangzhou Regenerative Medicine and Health Guangdong Laboratory, Guangzhou, China

**Keywords:** toll-like receptors (TLRs), neurodegenerative disorders, post-COVID-19 syndrome, neuroinflammation, SARS-CoV-2

## Abstract

In the intricate realm of interactions between hosts and pathogens, Toll-like receptors (TLRs), which play a crucial role in the innate immune response, possess the ability to identify specific molecular signatures. This includes components originating from pathogens such as SARS-CoV-2, as well as the resulting damage-associated molecular patterns (DAMPs), the endogenous molecules released after cellular damage. A developing perspective suggests that TLRs play a central role in neuroinflammation, a fundamental factor in neurodegenerative conditions like Alzheimer’s and Parkinson’s disease (PD). This comprehensive review consolidates current research investigating the potential interplay between TLRs, their signaling mechanisms, and the processes of neurodegeneration following SARS-CoV-2 infection with an aim to elucidate the involvement of TLRs in the long-term neurological complications of COVID-19 and explore the potential of targeting TLRs as a means of implementing intervention strategies for the prevention or treatment of COVID-19-associated long-term brain outcomes.

## 1 Introduction

The emergence of the Coronavirus Disease 2019 (COVID-19) pandemic, instigated by the novel Severe Acute Respiratory Syndrome Coronavirus 2 (SARS-CoV-2) virus, has been a transformative event in the annals of global public health with a documented death of nearly 7 million (1). Beyond its immediate impact on respiratory health, the virus unleashed a series of long-term medical challenges, notably the Post-Acute Sequelae of SARS-CoV-2 infection (PASC), sometimes referred to as ‘Long COVID’, ‘Long-haul COVID’ or ‘post-COVID syndrome’, where affected individuals report persistent symptoms long after initial recovery (2–5). However, the impact of a prior SARS-CoV-2 infection on the progression of subsequent infections has been unclear (6). The prevailing clinical outcomes suggest that a combination of organ damage and systemic inflammation could be responsible for sustained health complications post-COVID-19, affecting the respiratory, cardiac, neurological, and musculoskeletal systems (2, 4, 5, 7). Among the myriads of sequelae reported, neurological manifestations have been particularly enigmatic and concerning (8–10). Acute COVID has been linked to neurodegenerative diseases like Parkinson’s disease (PD) and cognitive challenges, from ‘brain fog’ to early Alzheimer’s disease (AD) onset. Additionally, the post-COVID syndrome is associated with various psychiatric disorders, including newly diagnosed conditions like depression, anxiety, and post-traumatic stress disorder (PTSD) (3). This has prompted researchers to dive into the complex interplay between the virus, the central nervous system (CNS), and the intricate immune responses, including the role of Toll-like Receptors (TLRs) (11).

The interaction between TLRs and the CNS has gained increased importance due to the potential neurotropic properties of the SARS-CoV-2 virus, which has demonstrated a propensity for invading neural tissues (12–15). The neurodegenerative consequences observed in individuals following COVID-19 infection appear to exhibit parallels with pathways involving the activation of TLRs, a connection that may present potential therapeutic targets. Epidemiological data and clinical studies further reinforce the potential role of TLRs in COVID-19 neurological symptoms (16–18), pushing them to the forefront of therapeutic and diagnostic considerations. As the scientific community works to elucidate this intricate landscape, greater attention needs to be directed toward the repurposing of existing TLR modulators and investigating their potential to target TLR signaling as therapeutic agents for the management of PASC-related complications. In this comprehensive review, we embark on a journey to elucidate the intricate relationship between the SARS-CoV-2 virus, TLRs, and the resulting neurological sequelae. By exploring current evidence, clinical findings, and therapeutic innovations, we aim to provide a roadmap for understanding, diagnosing, and potentially treating the neurological shadows cast by the COVID-19 infection.

## 2 Neurotropism of SARS-CoV-2

The emergence of SARS-CoV-2 heralded an unprecedented era in global health, largely dominated by its profound respiratory implications, with symptoms ranging from mild cough to severe respiratory distress (3, 19). Yet, as the pandemic progressed, accumulating evidence suggested that the virus has neurotropic properties, with the potential to invade and affect both the CNS and the peripheral nervous system (PNS) (20, 21). The occurrence rate of neurological symptoms following infection with SARS-CoV-2, collectively referred to as “Neuro-COVID,” varies considerably across studies and is infrequently attributed to the direct impacts of the virus (22, 23). The implications of this neurotropism have profound ramifications, as neurological manifestations have been reported in a significant proportion of COVID-19 patients, further complicating the clinical spectrum of the disease.

### 2.1 Routes of SARS-CoV-2 neuroinvasion

The SARS-CoV-2 demonstrates multiple transmission routes, prominently through respiratory fluids, underscoring its high infectiousness (24, 25). Notably, the human oral cavity and saliva have been identified as significant reservoirs for the virus (26). SARS-CoV-2 employs host entry factors such as ACE2 and TMPRSS family members (TMPRSS2 and TMPRSS4) to facilitate infection (27–29). Research has suggested that SARS-CoV-2 may travel retrogradely from peripheral regions to the CNS via olfactory sensory neurons or other neural pathways (27, 30). However, this proposed olfactory nerve route to brain infection remains controversial and lacks definitive evidence (31). Despite this, alternative pathways from the nasal cavity to the brain have been proposed, including vascular routes, CSF spaces, and the nervus terminalis system (31, 32). In humans, no longitudinal studies have mapped the timeline of neuro-invasion of SARS-CoV-2; only the end results have been documented (33, 34).

SARS-CoV-2 has been found in the olfactory epithelium, predominantly in sustentacular cells (35, 36). Studies using a rhesus monkey model have shown that SARS-CoV-2 can be transported to the CNS via the olfactory route following intranasal inoculation, inducing inflammatory cytokines and pathological lesions in the CNS (36–38). Observations in lethal COVID-19 cases indicate the virus might cross the neural-mucosal interface in the olfactory mucosa, leading to neurological diseases (36, 39). SARS-CoV-2 invades host cells primarily by binding to the ACE2 receptor on respiratory epithelial cell surfaces (25, 40, 41). Some patients have shown viral RNAs presence in the brain, particularly in the endothelial cells of the brainstem, thalamus, and hypothalamus, with occasional detection in the cerebral cortex and CSF (39, 42, 43). Other studies have reported the virus in leukocytes crossing the blood–brain barrier (BBB) or entering via endothelial cells of blood vessels, often referred to as a “Trojan horse” mechanism (44–47). Additionally, the virus can disrupt the endothelial glycocalyx and BBB integrity, facilitating its entry into the CNS (48). This disruption can lead to neurovascular dysfunction, as evidenced by increased markers of endothelial damage and coagulation abnormalities in Long COVID patients (46). Further, a novel entry route involving the CD147-spike protein has been identified, enabling the virions to enter host cells through clathrin-independent endocytosis, offering a new mechanism for SARS-CoV-2 infection and suggesting potential therapeutic strategies for COVID-19 (49). The involvement of CD147 in SARS-CoV-2 infection has also been questioned due to evidence indicating that CD147 does not have the capacity to bind to the spike protein (50).

The neurological symptoms seen in many COVID-19 patients suggest significant CNS penetrance by SARS-CoV-2 (39, 51, 52). Among the seven coronaviruses that infect humans, at least two endemic strains have demonstrated the ability to enter and persist in the CNS (39, 53, 54). The presence of SARS-CoV-2 RNA in the olfactory mucosa and neuroanatomical areas receiving olfactory tract projections implies possible neuroinvasion via axonal transport (43, 55, 56). Collectively, these findings indicate that SARS-CoV-2 can invade the CNS via the neural-mucosal interface and olfactory tract, contributing to the neurological symptoms observed in COVID-19. The exact mechanisms of SARS-CoV-2 neuroinvasion remain under investigation, with proposed routes including hematogenous spread and transneuronal dissemination through the olfactory nerve. The virus’s interaction with ACE2 receptors in various CNS cells, including neurons and glial cells, further underscores its potential to breach the BBB and infect the CNS. Understanding these pathways is crucial for developing strategies to mitigate the neurological impacts of COVID-19.

### 2.2 Immune reactions in the CNS during COVID-19 and PASC

The immune response to COVID-19 and PASC is complex, involving both innate and adaptive mechanisms that contribute to the pathology and persistence of neurological symptoms (57–59). Understanding these immune reactions is critical for developing targeted therapies and improving patient outcomes. During the acute phase of COVID-19, the CNS can be affected directly by viral invasion or indirectly through systemic inflammation (60–62). SARS-CoV-2 may enter the CNS via the olfactory nerve and bloodstream or by infecting endothelial cells of the BBB, leading to neuroinflammation (43, 63). The initial innate immune response involves the activation of pattern recognition receptors (PRRs) such as TLR3, TLR7, RIG-I, and MDA5, which detect viral RNA and trigger the production of type I interferons and other pro-inflammatory cytokines (64–67). This robust immune response is intended to control viral replication but can lead to a cytokine storm characterized by elevated levels of cytokines such as interleukin (IL)-6, tumor necrosis factor (TNF)-*α*, and IL-1β, exacerbating inflammation and tissue damage (68–70). In severe cases, this can result in widespread neuroinflammation, microglial activation, and neuronal damage, manifesting as acute neurological symptoms like anosmia, headache, and cognitive impairment (70–72). Queiroz et al. (73) examined the involvement of the cyclic GMP-AMP synthase (cGAS)-stimulator of interferon genes (STING) pathway in COVID-19 severity and ‘Long COVID’. The research analyzed blood samples from 148 individuals, including those with acute and ‘Long COVID’. It was observed that in acute COVID-19 cases, higher expression levels of cGAS, STING, and cytokines [interferon (IFN)-*α*, TNF-α, IL-6] were present in patients with severe disease compared to those with non-severe manifestations. Similarly, Long COVID was associated with elevated levels of cGAS, STING, and IFN-α, indicating a persistent inflammatory response. This pathway’s activation appears to contribute to an intense systemic inflammatory state in severe COVID-19 and, after infection resolution, may induce autoinflammatory conditions in various tissues, resulting in Long COVID.

The adaptive immune system also plays a significant role in the pathogenesis of neuro-PASC (58, 59). Studies have shown that patients with neuro-PASC have elevated levels of inflammatory markers and immune cells in the CSF, including exhausted CD4+ T cells, border-associated macrophages, microglia, and granulocytes (74, 75). The presence of exhausted T cells in the CSF suggests an ongoing but ineffective immune response, potentially due to the absence of active viral replication, as most neuro-PASC CSF samples are negative for SARS-CoV-2 (2, 23, 58). Elevated levels of IL-12 in the CSF of neuro-PASC patients further indicate a skewed immune response, which may contribute to sustained neuroinflammation and tissue damage (74). There is evidence of an increased frequency of B and T cells in the CNS of neuro-PASC patients (75, 76). Some of these B cells produce IgG antibodies with anti-neural reactivity, suggesting a possible autoimmune component to the disease (75, 77). This autoimmune response may be driven by molecular mimicry, where viral antigens resemble host neural antigens, leading to cross-reactivity and autoimmunity (78). Furthermore, studies have found elevated levels of autoantibodies in patients with ‘Long COVID,’ which may contribute to persistent neurological symptoms by targeting CNS tissues and exacerbating inflammation (2).

The long-term implications of these immune reactions in the CNS are significant, with potential links to neurodegenerative diseases (79). For example, the chronic neuroinflammation observed in neuro-PASC patients may accelerate or trigger conditions like AD, especially in older individuals (80). The similarities between the neuropathological features of COVID-19 and AD, such as the involvement of the orbitofrontal cortex and parahippocampal gyrus, highlight the need for further research in this area (33, 81). Future studies should focus on elucidating the precise mechanisms of immune dysregulation in neuro-PASC and identifying biomarkers for early diagnosis and targeted therapy. Understanding the balance between protective and pathological immune responses will be crucial for developing interventions that mitigate long-term neurological sequelae while effectively controlling viral replication. Overall, the immune response in the CNS during COVID-19 and PASC involves a complex interplay of innate and adaptive mechanisms that can lead to persistent neuroinflammation and neurological symptoms. Ongoing research is essential to understand these processes fully and to develop effective treatments for those suffering from ‘Long COVID.’

### 2.3 Neurological manifestations directly attributed to COVID-19

The neurological manifestations directly attributed to COVID-19 encompass a broad spectrum impacting the CNS (82). The neurological manifestations of COVID-19 are diverse and can significantly impact patient outcomes (83). These manifestations can be classified into CNS symptoms like dizziness, headaches, impaired consciousness, acute cerebrovascular disease, ataxia, seizures, and PNS, including taste and smell impairments, vision disturbances, and nerve pain (21).

Among the CNS manifestations, encephalitis, an inflammation of the brain observed in COVID-19 patients, presents with symptoms such as headache, fever, confusion, seizures, and focal neurological deficits. Severe cases can lead to acute necrotizing hemorrhagic encephalopathy and rhombencephalitis, conditions confirmed by magnetic resonance imaging (MRI) showing characteristic brain changes (84–86). SARS-CoV-2 has occasionally been found in CSF, indicating direct viral invasion and immune-mediated mechanisms (2). Acute Disseminated Encephalomyelitis (ADEM), a rare inflammatory condition affecting the brain and spinal cord, has also been reported, presenting with rapid onset of neurological symptoms such as ataxia, motor deficits, and altered consciousness, often treated with immunosuppressive therapy (87, 88). COVID-19 increases the risk of cerebrovascular events, including ischemic and hemorrhagic strokes, linked to the virus-induced prothrombotic state, endothelial damage, and cytokine storm (89, 90). Strokes in COVID-19 patients, seen in 2.5 to 5% of cases, typically reveal large vessel occlusions or hemorrhagic lesions on neuroimaging (90, 91).

Among the PNS manifestations, Guillain-Barré Syndrome (GBS), characterized by acute flaccid paralysis, is associated with COVID-19, often presenting with progressive weakness and sensory disturbances following initial respiratory symptoms, likely due to immune responses misdirected at peripheral nerves (92). COVID-19 also causes cranial neuropathies, including anosmia and ageusia, resulting from viral invasion of the olfactory epithelium and spread to the olfactory bulb (93, 94). Cases of oculomotor nerve palsy and optic neuritis, causing double vision and vision loss, have been documented (95, 96). Muscle involvement ranges from mild myalgias to severe rhabdomyolysis, leading to muscle breakdown and potential kidney damage, marked by elevated creatine kinase levels (97, 98). Additionally, COVID-19 has been linked to demyelinating disorders, with MRI showing lesions and a hyperinflammatory state potentially exacerbating or triggering these conditions (99, 100). Furthermore, the post-infectious implications are only beginning to be understood. Persistent cognitive deficits, termed “brain fog,” along with fatigue and mood disorders, have been reported in PASC cases, underscoring the lasting impact of the virus on the CNS (101, 102). The neurotropic nature of SARS-CoV-2 has expanded the scope of COVID-19 beyond a respiratory illness, bringing to light the intricate and profound interplay between the virus and the nervous system. As we continue to grapple with the pandemic’s ramifications, understanding the neurological facets of COVID-19 will be pivotal in devising effective therapeutic and rehabilitative strategies.

## 3 TLRs in CNS: balancing immune defense and neuroinflammation

**Figure 1:**
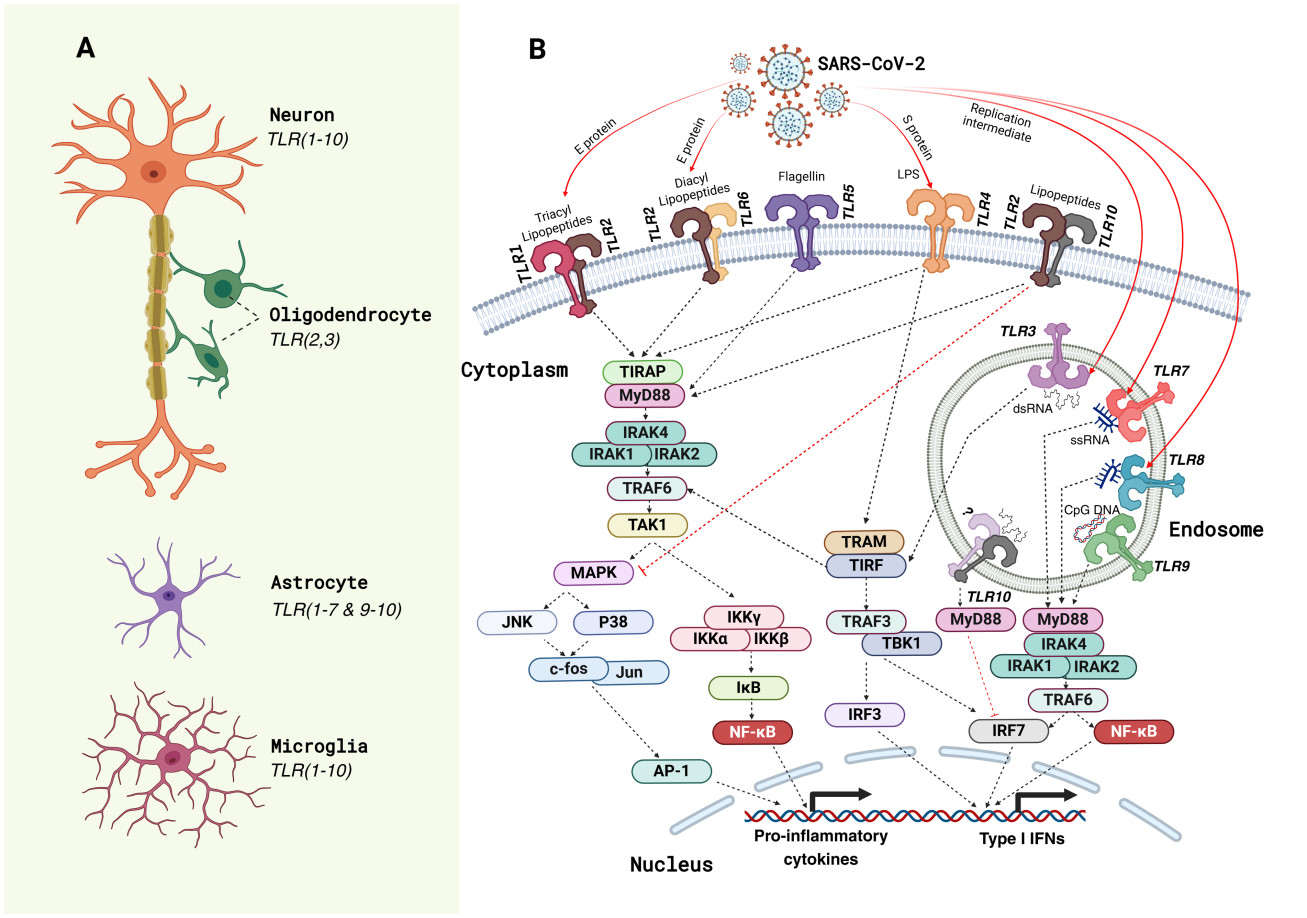
Toll-like receptor (TLR) expression and SARS-CoV-2-induced TLR signaling pathways in neural cells. **(A)** The schematic illustrates the expression profile of Toll-like receptors (TLRs) in key neural cell types of the central nervous system (CNS) in humans. Neurons predominantly express almost all TLRs (1–10); oligodendrocytes express TLR2 and TLR3; astrocytes express all TLRs except TLR8, whereas microglia, the resident immune cells of the CNS, express the broadest range of TLRs (TLR1–10) (179), underscoring their critical role in innate immune surveillance and neuroinflammatory responses. **(B)** TLR signaling pathways activated by SARS-CoV-2 components are depicted. The viral proteins (spike [S], envelope [E], and nucleocapsid [N]) and associated pathogen-associated molecular patterns (PAMPs) such as lipopolysaccharides (LPS) and double-stranded RNA (dsRNA) interact with specific TLRs on the cell membrane (e.g., TLR1, TLR2, TLR4, TLR5, and TLR6) and within endosomal compartments (e.g., TLR3, TLR7, TLR8, and TLR9). These interactions activate downstream signaling cascades through adaptor proteins like myeloid differentiation primary response protein 88 (MyD88) and TIR domain-containing adapter inducing IFNβ (TRIF). Subsequent recruitment of kinases such as interleukin (IL)-1 receptor-associated kinase (IRAK)-1/4, tumor necrosis factor receptor-associated factor (TRAF6), and TANK-binding kinase 1 (TBK1) leads to the activation of transcription factors nuclear factor (NF)-κB, activator protein 1 (AP-1), and interferon regulatory factors (IRFs), which translocate to the nucleus to induce the expression of Type I interferons (IFNs) and pro-inflammatory cytokines. These signaling events mediate antiviral immunity but may contribute to the hyperinflammatory responses observed in COVID-19 neuropathology. TIR domain-containing adapter protein (TIRAP); transforming growth factor-activated kinase (TAK); mitogen-activated protein kinase (MAPK); c-Jun N-terminal kinase (JNK); inhibitor of kappa light polypeptide gene enhancer in B-cell kinase (IKK); inhibitor of nuclear factor kappa B (IĸB); Trif-related adapter molecule (TRAM); single-stranded RNA (ssRNA).

TLRs are crucial components of the innate immune system, acting as primary detectors of pathogenic threats (103, 104). These receptors recognize conserved molecular structures known as pathogen-associated molecular patterns (PAMPs), which are essential for microbial survival but absent in host cells (105, 106). TLRs are type I transmembrane proteins with extracellular leucine-rich repeat (LRR) domains for PAMP recognition and intracellular Toll/interleukin-1 receptor (TIR) domains for initiating immune signaling pathways (105, 107). TLRs are classified into cell surface TLRs (e.g., TLR1, TLR2, TLR4, TLR5, TLR6, and TLR10), which detect microbial membrane components, and endosomal TLRs (e.g., TLR3, TLR7, TLR8, and TLR9), which recognize microbial nucleic acids (103, 108). Upon detecting PAMPs, TLRs dimerize and recruit adaptor proteins like MyD88 and TRIF, forming complexes that activate downstream signaling pathways (107, 109). The MyD88-dependent pathway primarily induces pro-inflammatory cytokines through NF-κB and MAPKs, while the TRIF-dependent pathway promotes type I interferons and other inflammatory responses (Figure 1) (103, 110, 111). These pathways are essential for an effective immune response and linking innate and adaptive immunity.

TLRs are vital in dendritic cell (DC) maturation and crucial for antigen presentation and T cell activation (103, 112). Activated DCs express higher levels of co-stimulatory molecules and MHC, enhancing their ability to initiate adaptive immune responses (113, 114). TLRs are also key regulators in macrophage function and inflammatory responses. Sanjuan et al. (115) demonstrated that TLR activation during phagocytosis mobilizes autophagy proteins to the phagosome, thereby enhancing phagosome maturation and pathogen destruction in macrophages. Recent studies underscore the critical roles of TLRs in modulating macrophage function and polarization. TLR7 agonists have been shown to repolarize M2 macrophages to the pro-inflammatory M1 phenotype, thereby enhancing phagocytosis, inflammation, and antigen presentation (116). Similarly, Vidyarthi et al. (117) elucidated the importance of TLR3 signaling in skewing M2 macrophages to the M1 subtype, both *in vitro* and *in vivo*. Further emphasizing the versatility of TLRs, Geng et al. (118) reported that TLR4 coordinates with the mechanical sensor Piezo1 to activate the CaMKII-Mst1/2 axis, driving macrophages to execute essential host defense functions. Collectively, these findings highlight the multifaceted roles of TLRs in macrophage biology, presenting them as promising therapeutic targets for enhancing immune responses.

Similarly, Veneziani et al. (119) demonstrated that TLR8 agonists effectively activate NK cells, particularly the CD56bright subset, by enhancing their proliferation, cytokine production, and cytotoxicity, which are vital in the tumor microenvironment. This activation promotes a pro-inflammatory phenotype, improving interactions between NK cells and dendritic cells (DCs) and boosting tumor antigen presentation and NK cell-mediated cytotoxicity against tumor cells. The study also highlighted that the TLR8 agonist R848 uniquely enhances CD56brightCD16− NK cell functions, especially when combined with suboptimal cytokine levels like IL-2 and IL-12, a specificity not shared by TLR3 and TLR9 agonists. This activation, exclusive to TLR8 and not TLR7, significantly increases IFN-*γ* and TNF-*α* production, suggesting TLR8 agonists as promising agents for cancer immunotherapy (119). Similarly, Noh et al. (120) reviewed the critical role of TLRs in NK cells, noting their ability to recognize pathogens and induce strong immune responses. TLR agonists enhance NK cell cytotoxicity and cytokine production, improving the efficacy of immunotherapies, including monoclonal antibody treatments for cancer.

Additionally, TLRs are expressed in non-immune cells like epithelial and endothelial cells, contributing to immune responses by producing cytokines and chemokines (121, 122). However, dysregulated TLR activation can lead to chronic inflammation and autoimmune diseases by recognizing self-derived damage-associated molecular patterns (DAMPs) (123, 124). Thus, TLR signaling must be tightly regulated to balance protective immunity and prevent pathological inflammation. Overall, TLRs are fundamental to innate immunity, recognizing PAMPs to initiate immune responses and shaping adaptive immunity. Understanding TLR signaling mechanisms is key to developing therapies for infectious, inflammatory, and autoimmune diseases.

The CNS, once considered an immune-privileged site, is now recognized as a dynamic environment where immune surveillance and responses occur (125, 126). TLRs are pivotal in this context, primarily expressed by microglia, the resident immune cells of the brain, but also found in astrocytes and other CNS cell types (127–129). Microglia express a wide array of TLRs, enabling them to detect and respond to diverse pathogens and cellular stress signals (130, 131). For instance, TLR4 recognizes lipopolysaccharides (LPS) from bacterial cell walls, while TLR3 detects viral double-stranded RNA (105, 132, 133). A significant aspect of TLR function in the CNS is their interaction with the inflammasome, particularly the NLR family pyrin domain containing 3 (NLRP3) inflammasome (134, 135). The inflammasome is a multi-protein complex that processes pro-IL-1β and pro-IL-18 into their active forms via caspase-1 (136, 137). This process requires a two-signal model: the first signal involves TLR-mediated transcription of pro-IL-1β and pro-IL-18, and the second signal, often provided by DAMPs like ATP, leads to inflammasome assembly and activation (127, 134).

Upon TLR activation, microglia release various pro-inflammatory mediators, including IL-1β, which plays a central role in the pathogenesis of numerous neurodegenerative diseases and CNS injuries (138, 139). For example, TLR4 activation in microglia can lead to significant neuroinflammation, contributing to conditions such as AD, multiple sclerosis, and stroke (128). Astrocytes also contribute to TLR-mediated responses in the CNS, albeit to a lesser extent than microglia. They express a limited repertoire of TLRs and can respond to inflammatory stimuli by producing cytokines and chemokines that influence microglial activity and overall CNS inflammation (17, 130). However, the exact role of inflammasomes in astrocytes remains less precise compared to microglia (91, 140). TLR signaling in the CNS is a double-edged sword (129, 141). While it is essential for defending against infections and maintaining homeostasis, excessive or prolonged TLR activation can lead to chronic neuroinflammation and subsequent neuronal damage (17). This is particularly relevant in neurodegenerative diseases where TLR activation can exacerbate disease progression by promoting sustained inflammation. Understanding the nuanced roles of TLRs in the CNS could pave the way for targeted therapies that modulate immune responses, aiming to protect against infections while minimizing harmful inflammation in neurodegenerative and other CNS disorders.

## 4 Involvement of TLRs in neurodegenerative disease pathogenesis

TLRs are key players in the pathogenesis of various neurodegenerative diseases due to their role in mediating inflammatory responses and other functions such as microglial activation and synaptic dysfunction (142–146). These receptors are expressed not only in immune cells but also in neurons, astrocytes, and microglia within the CNS, where they can recognize both PAMPs and DAMPs (130, 143).

TLRs play a significant role in the pathogenesis of AD through their involvement in neuroinflammation and the innate immune response (147). In AD, TLRs are crucial in recognizing amyloid-beta (Aβ) plaques and tau protein tangles, which are hallmarks of the disease (148). For instance, TLR4 has been implicated in recognizing Aβ plaques (148–150). Upon activation, TLR4 triggers signaling pathways that lead to the production of pro-inflammatory cytokines such as TNF-*α*, IL-1β, and IL-6, contributing to neuroinflammation and neuronal damage (107, 151). This chronic inflammation can exacerbate the pathology of AD, suggesting that TLRs are significant contributors to the disease’s progression (152). Moreover, TLR2 and TLR9 also play roles in AD by enhancing the clearance of Aβ and modulating neuroinflammatory pathways (153).

A detailed examination of TLRs in AD draws on multiple studies and offers a comprehensive understanding of their contributions to the disease (Table 1). TLR4 is one of the most studied TLRs in relation to AD (153–157). They are localized on the surface of microglia, the resident immune cells in the brain, and are responsible for recognizing LPS and Aβ peptides, both associated with inflammatory responses in AD (139, 157). Research has shown that genetic polymorphisms in TLR4 can influence AD occurrence. Specifically, the Asp299Gly polymorphism of TLR4 has been associated with a decreased risk of late-onset AD (LOAD). This polymorphism attenuates inflammatory response due to reduced receptor signaling (155). Further, in this study, which involved the Italian population (277 LOAD patients), the frequency of the minor TLR4 299Gly allele was significantly higher in controls (300 cognitively healthy controls) compared to LOAD patients, and this variant was suggested to be protective toward the development of LOAD (155). Studies have demonstrated that TLR4 expression is significantly upregulated in the brains of AD patients (158, 159). This upregulation correlates with the presence of Aβ plaques and neurofibrillary tangles, the pathological hallmarks of AD. Further, the studies suggest that variations in TLR4 can affect its expression and function, thereby modulating the risk of developing AD (154). For instance, Zhang et al. (153) found increased expressions of TLR4 on peripheral blood mononuclear cells from AD patients, indicating a systemic inflammatory response in addition to the local brain inflammation. TLR2, like TLR4, is involved in recognizing Aβ and plays a role in mediating inflammatory responses in AD (153). Increased expression of TLR2 has been observed in microglia surrounding Aβ plaques. The activation of TLR2 by Aβ leads to the production of pro-inflammatory cytokines, contributing to the neuroinflammatory milieu characteristic of AD (153).

**Table 1:** Summary of TLR involvement in neurodegenerative diseases and other CNS disorders.

TLR type	Role in NDs	Reference
Alzheimer’s disease (AD)		
TLR2	TLR2 is increased in peripheral blood mononuclear cells in LOAD.	(153)
	TLR2 is significantly elevated in the hippocampus and cortex of AD patients.	(162)
TLR3	TLR3 expression by brain microglia is upregulated with increasing AD pathology.	(237)
TLR4	TLR4 contributes to AD neuroinflammation via an amyloid-independent differentiation process.	(156)
	Single nucleotide polymorphism (SNP) of the +896A/G TLR4 gene is associated with AD.	(154)
	TLR4 expression is decreased and vulnerable to degeneration in AD.	(157)
	TLR4 gene 299Gly allele is related to a decreased risk of sporadic late-onset AD (LOAD).	(155)
	TLR2 and TLR4 are increased in peripheral blood mononuclear cells in LOAD.	(153)
TLR5	TLR5 levels might rise in the brain areas of patients with AD. Rare coding variants in human TLR5 could be linked to a lower risk of developing the disease AD	(163)
TLR9	TLR9 p.317D mutation increases AD risk by disrupting innate immunity.	(161)
	TLR9 rs187084 polymorphism contributes to decreased LOAD risk.	(160)
Parkinson’s disease (PD)		
TLR2	TLR2 is required for enhancing exosomal α-synuclein transmission by reactive microglia.	(165)
	Increased TLR2 promotes α-synuclein aggregation in autophagy/lysosomal pathways.	(166)
	TLR2 is involved in the microglial-mediated responses for PD development.	(164)
	TLR2 is increased in circulating monocytes, and TLR4 is also increased in B cells.	(168)
	Blood leukocyte TLR2 responses are impaired.	(167)
	TLR2-induced cytokine production is controlled by TLR10.	(173)
TLR4	Increased TLR4 triggers elevated production of IL-1β in multiple PD brain regions.	(171)
	HMGB1-TLR4 axis and downstream factors positively correlate with PD stage, duration, and therapeutic outcomes.	(169)
	TLR4 is increased in the substantia nigra of PD patients.	(170)
	TLR4 is increased in circulating monocytes, and TLR4 is also increased in B cells.	(168)
TLR7/8	Blood leukocyte TLR7/8 responses are impaired	(167)
TLR9	Rs352140 T allele of the TLR9 gene is related to the reduced PD risk.	(172)
TLR10	TLR10 plays a role in controlling TLR2-induced cytokine production, and increased TLR10 is related to reduced PD severity.	(173)
Other CNS diseases		
TLR2	Urinary TLR2 stimulants and serum-soluble TLR2s are upregulated in MS patients.	(176)
	TLR2 is increased in the post-mortem spinal cord of ALS patients.	(177)
TLR4	TLR4 acts as a genetic modifier for HD progression.	(178)
	TLR4 is increased in the post-mortem spinal cord of ALS patients.	(177)

This table presents an overview of the roles played by various Toll-like receptors (TLRs) in different neurodegenerative diseases and other central nervous system (CNS) disorders. The diseases covered are Alzheimer’s Disease (AD), Parkinson’s Disease (PD), Multiple Sclerosis (MS), Amyotrophic Lateral Sclerosis (ALS), and Huntington’s Disease (HD). Based on findings from various studies, the table highlights how changes in TLR expression and function contribute to disease mechanisms and progression.

Recent research highlights the potential involvement of TLR9 in AD pathogenesis, particularly its role in the immune system’s response to Aβ deposits. TLR9 is known for recognizing unmethylated CpG DNA from pathogens, triggering an immune response. Studies have suggested TLR9 signaling may be implicated in Aβ clearance, an AD hallmark (160). In a study focusing on a large Han Chinese population, a significant association was found between a polymorphism in the TLR9 gene (rs187084) and a decreased risk of LOAD. This polymorphism was linked to higher expression levels of TLR9 in peripheral blood monocytes, suggesting that enhanced TLR9 activity might facilitate the clearance of Aβ, thus reducing AD risk (160). Moreover, research involving a Flanders-Belgian family identified a novel variant (p.E317D) in TLR9 that co-segregates with early-onset AD (EOAD) in an autosomal dominant manner. This variant caused a 50% reduction in TLR9 activation in an NF-κB luciferase assay, indicating a loss-of-function mutation. The resulting cytokine profile from human peripheral blood mononuclear cells (PBMCs) upon TLR9 activation showed an anti-inflammatory response that promoted phagocytosis of Aβ42 oligomers in human iPSC-derived microglia. This finding underscores the protective role of TLR9 signaling in AD pathogenesis and suggests that loss-of-function mutations may disrupt peripheral-central immune crosstalk, leading to increased neuroinflammation and pathogenic protein aggregates in AD (161). TLR3, another member of the TLR family, is also implicated in the pathogenesis of AD through its role in recognizing double-stranded RNA and activating immune responses. Specifically, TLR3-induced neuroinflammation can contribute to neuronal damage and the progression of AD pathology (153). For instance, TLR3 signaling has been shown to upregulate the expression of genes that modulate the production and clearance of Aβ, suggesting a potential mechanism by which TLR3 contributes to AD progression (153). The involvement of TLRs in AD extends beyond genetic associations. Mechanistically, TLR activation triggers downstream signaling pathways, such as NF-κB, which promotes the transcription of pro-inflammatory cytokines like TNF-*α*, IL-1β, and IL-6. The role of TLR4 in mediating these responses has been particularly well-documented, with studies showing that TLR4-deficient mice exhibit reduced microglial activation and cytokine production in response to Aβ (157). Research findings also suggest a role for TLR2 in AD pathogenesis (153). TLR2 has been shown to be upregulated in the prefrontal cortex and hippocampus of AD patients and 5XFAD mouse models (162). Furthermore, TLR5 has also emerged as a potential modulator of AD pathology. Genetic analyses conducted in human AD brains have suggested that rare protein-coding variants in TLR5 may be associated with a reduced risk of AD (163).

In PD, the misfolded protein alpha-synuclein (*α*-syn) accumulates and activates TLR2 and TLR4 in microglia. This activation leads to the production of inflammatory mediators and reactive oxygen species (ROS), which contribute to the neurodegenerative process. Studies have shown that TLR2 expression is significantly increased in the substantia nigra and anterior cingulate cortex of PD patients, correlating with disease severity (164). This upregulation correlates with the accumulation of α-syn, a protein that aggregates and forms Lewy bodies, characteristic of PD (165). Experimental models have demonstrated that TLR2 deficiency can reduce alpha-synuclein accumulation and neuroinflammation, highlighting its role in PD pathogenesis (143). TLR2 is found in microglia, neurons, and within Lewy bodies, indicating its widespread involvement in the pathogenesis of PD (166). In addition to its expression in the CNS, TLR2 is also present in peripheral immune cells. Blood monocytes from PD patients exhibit altered responses to TLR2 activation, with decreased production of cytokines such as TNF-*α*, IL-1β, IL-6, and IL-10 compared to controls. This impaired cytokine response suggests a dysregulation of TLR2 and TLR7/8-mediated immune functions in PD (167). Further, TLR2 and TLR4 are also significantly involved in PD, with increased expression in the blood and brain leading to microglial activation, inflammatory cytokine production, and neurodegeneration, indicating potential therapeutic targets to mitigate inflammation and slow disease progression (168).

TLR4 plays a pivotal role in the pathogenesis of PD by mediating inflammatory responses and contributing to neurodegeneration. Yang et al. (169) demonstrated that TLR4 expression is significantly elevated in the serum of PD patients compared to healthy controls, and this elevation correlates with disease progression, duration, and poor responses to drug treatments, highlighting TLR4’s critical involvement in PD pathology. This finding underscores the systemic nature of TLR4-related inflammation and its potential as a biomarker for disease severity (169). Shin et al. (170) expanded on this by showing that prothrombin kringle-2 (pKr-2), a non-toxic fragment of prothrombin, induces TLR4 expression in microglia within the substantia nigra of PD patients. The study suggests that inhibiting pKr-2-induced TLR4 activation could be a protective strategy against dopaminergic neuron loss in PD (170). Further supporting these findings, Kouli et al. (171) reported that TLR4 activation by *α*-syn aggregates significantly contributes to the chronic neuroinflammatory environment observed in PD. They noted increased TLR4 expression in both the CNS and peripheral immune cells of PD patients, emphasizing its role in exacerbating neurodegeneration. The study by Miri et al. (172) found that the rs352140T polymorphism in the TLR9 gene acts as a protective factor against PD in the northern Iranian population. This single-nucleotide polymorphism (SNP) was significantly associated with a reduced risk of PD, suggesting that individuals carrying the rs352140T allele have a lower likelihood of developing the disease. Additionally, the study highlighted that the TLR9 rs352140T allele could be considered a potential prognostic marker and therapeutic target for PD, emphasizing the importance of genetic factors in modulating immune responses and disease susceptibility (172). The study by da Rocha Sobrinho et al. found that TLR10 plays a crucial role in controlling TLR2-induced cytokine production in monocytes from PD patients. The elevated expression of TLR10 on monocytes was associated with reduced production of pro-inflammatory cytokines, suggesting a protective mechanism against PD progression (173).

Multiple sclerosis (MS) is characterized by chronic inflammation and demyelination in the CNS. TLR2 and TLR4 activation in response to myelin debris and other DAMPs leads to pro-inflammatory cytokines and chemokines, promoting immune cell infiltration and further demyelination. The complex role of TLRs in MS is evidenced by varying effects of TLR deficiencies, which can either ameliorate or exacerbate the disease in experimental models (143). TLR3 and TLR9 have also been implicated in MS, with their activation contributing to neuroinflammation and neurodegeneration (174, 175). A recent study found that soluble TLR2 (sTLR2) levels were significantly elevated in the serum of MS patients during both relapse and remission compared to healthy controls, suggesting sTLR2 as a potential biomarker for MS (176). Further, TLR2 stimulants were significantly higher in the urine of MS patients than in healthy controls. This finding indicates that MS patients are exposed to higher levels of TLR2 ligands, possibly contributing to disease activity and progression (176).

Amyotrophic lateral sclerosis (ALS) involves selective motor neuron degeneration, and TLRs have been implicated in its pathogenesis. TLR2 and TLR4 levels are elevated in the spinal cord of ALS patients, particularly in microglia and astrocytes. These receptors mediate neuroinflammatory responses that exacerbate motor neuron death. In mouse models of ALS, the deletion of TLR4 has been shown to improve survival and reduce neurological deficits, underscoring the detrimental role of TLR-mediated inflammation in ALS (143). A research study findings demonstrate heightened expression of TLR2, TLR4, the receptor for advanced glycation end products (RAGE), and high mobility group box 1 (HMGB1) in reactive glia in the ALS spinal cord, suggesting the activation of TLR/RAGE signaling pathways resulting in the injury of motor neurons (177). Vuono et al. (178) observed a significant increase in the number of cells expressing TLR4 in the striatum of postmortem brain samples from Huntington’s disease (HD) patients compared to controls. This finding suggests that TLR4-mediated inflammatory responses are involved in HD pathogenesis. The study also identified that specific TLR4 SNPs were associated with changes in motor progression in HD patients. Specifically, the rs1927911 and rs1927914 polymorphisms were linked to a faster rate of motor decline, indicating that genetic variations in TLR4 may influence the clinical progression of HD (178).

Across these diseases, TLRs function as critical modulators of neuroinflammation and neuronal degeneration. Their activation often results in the production of pro-inflammatory cytokines and reactive oxygen species, contributing to a chronic inflammatory environment that accelerates disease progression. However, the precise role of each TLR may vary, as TLR-mediated signaling can have context-dependent effects in different disease states, potentially resulting in either detrimental or beneficial outcomes. In AD, TLR4 and TLR2 play pivotal roles in identifying and eliminating Aβ plaques and tau tangles. On the other hand, these receptors have the capacity to initiate chronic inflammation, which aggravates the progression of the disease. In a similar manner, in PD, the activation of TLR2 and TLR4 by *α*-synuclein aggregates prompts microglial activation and oxidative stress, thereby expediting neurodegeneration. Whereas, TLRs such as TLR9 and TLR10 function as protective factors by mitigating the production of pro-inflammatory cytokines. However, in MS, TLR9 as well as other TLRs such as TLR2, TLR3, TLR4 mediate inflammatory cascades linked to demyelination. Additionally, TLR-mediated signaling pathways are implicated in ALS and HD, highlighting their systemic contributions to disease-specific inflammatory processes and progression. Understanding the distinct roles of TLRs in these diseases offers valuable insights into the complex interplay between the immune system and neurodegeneration, providing a foundation for the development of targeted therapies aimed at modulating TLR signaling to mitigate neuroinflammation and slow disease progression. Modulating TLR activity could help balance the beneficial and detrimental effects of inflammation. For example, TLR4 inhibitors might reduce neuroinflammation in AD and PD, while strategies to enhance TLR-mediated clearance of protein aggregates could be beneficial in early disease stages. Nevertheless, therapeutic interventions must be meticulously tailored to prevent undesired suppression of protective immune responses (179).

## 5 Involvement of TLRs in the pathogenesis of SARS-CoV-2 infection

TLRs play significant roles in recognizing pathogens and initiating immune responses (104). Several studies have investigated the involvement of TLRs in neuroinflammation induced by SARS-CoV-2 infection, providing insights into their mechanisms and potential therapeutic targets (180–183). SARS-CoV-2, the virus responsible for COVID-19, interacts with various TLRs to trigger immune responses (182). TLR2, for instance, has been identified as a receptor that recognizes the envelope (E) protein of SARS-CoV-2 (184). This interaction leads to the activation of downstream signaling pathways, resulting in the production of pro-inflammatory cytokines (141). TLR4 is also implicated in SARS-CoV-2 infection, where it recognizes viral components and contributes to the inflammatory response (185). Both TLR2 and TLR4 play pivotal roles in mediating neuroinflammation during SARS-CoV-2 infection (183, 186). TLR2, expressed on microglia and neurons, is activated by SARS-CoV-2, leading to the release of inflammatory cytokines and the promotion of neuroinflammation. This process is suggested to exacerbate neurodegenerative conditions such as AD and PD by increasing Aβ and *α*-syn aggregation (185). TLRs’ involvement in SARS-CoV-2 infection is also linked to their effects on the BBB. The inflammation induced by TLR activation can compromise the integrity of the BBB, allowing SARS-CoV-2 and immune cells to infiltrate the CNS. This breach is a critical step in the pathogenesis of COVID-19-related neurological symptoms, including encephalitis, cerebrovascular events, and neurodegeneration (141). Given their central role in mediating neuroinflammation, TLRs present potential therapeutic targets for mitigating SARS-CoV-2-induced neurological damage. Modulating TLR activity, either through agonists or antagonists, could help regulate the immune response and reduce the excessive inflammation that contributes to CNS pathology. This approach holds promise for preventing or treating the neuroinflammatory consequences of COVID-19.

Evidence from recent research provides comprehensive insights into the role of various TLRs in COVID-19 infection and severity, highlighting the intricate interplay between genetic variations, immune responses, and clinical outcomes (Table 2). Zheng et al. (187) demonstrated that TLR2 recognizes the SARS-CoV-2 E protein, initiating inflammatory signaling pathways and cytokine production, essential for the inflammatory response during *β*-coronavirus infections. Inhibition of TLR2 signaling significantly reduces key inflammatory cytokines and chemokines, such as IL-6, CXCL10, and MCP-1, and improves survival rates in SARS-CoV-2-infected mice, suggesting a potential therapeutic strategy to mitigate severe COVID-19 pathology. Building on this, Nayak et al. (188) reveal the minor deletion allele of the TLR2 rs111200466 polymorphism is inversely correlated with susceptibility to SARS-CoV-2 infection and mortality. This indicates that TLR2 genetic variants, specifically the rs111200466 polymorphism, may influence COVID-19 incidence and severity, warranting further validation in diverse populations. Alhabibi et al. (189) highlight TLR4’s critical role in the inflammatory response to SARS-CoV-2, with the minor alleles 299Gly (G) and 399Ile (T) significantly associated with severe COVID-19 and increased IL-6 levels, contributing to cytokine storm and higher mortality rates. They also identify TLR2 and TLR9 variants linked to increased risk and severity of COVID-19, specifically the TLR2 rs5743708 (G/A) and TLR9 rs5743836 (C/C) genotypes, which correlate with pro-inflammatory responses and disease progression.

**Table 2:** Roles of toll-like receptors (TLRs) in COVID-19 pathogenesis.

TLR type	Role of TLR in COVID-19	Reference
TLR2	TLR2 rs111200466 variant can protect against SARS-CoV-2 infections and mortality.	(188)
	TLR2 can recognize SARS-CoV-2 envelope protein to produce inflammatory cytokines.	(187)
	The response of TLR2 to SARS-CoV-2 may lead to a mass synthesis of pro-inflammatory mediators and contribute to hyperinflammation in severe COVID-19.	(190)
	TLR2 rs5743708 variants are genetic risk factors for COVID-19 severity.	(189)
	TLR2 may contribute to immunosuppression following hyperinflammation in COVID-19 by reducing IL-8 secretion.	(191)
TLR3	Systemic TLR3 level negatively correlates with impaired lung function and short/long-term neurological outcomes.	(193)
	TLR3 L412F polymorphism is a genetic risk factor for COVID-19 severity in males by inhibiting autophagy.	(192)
	TLR3 mutant rs3775291 positively correlates with SARS-CoV-2 susceptibility and mortality due to limited protective immune responses.	(194)
	Lower expression of TLR3 is associated with an unfavorable outcome in severe COVID-19 patients.	(195)
	TLR3 rs3775290 polymorphisms may be risk factors for vulnerability to COVID-19.	(196)
	Hyperactivation of TLR3 may lead to higher severity of COVID-19.	(197)
TLR4	The rs4986790 GG genotype of TLR4 affects COVID-19 severity by limiting the delivery of pro-inflammatory cytokines.	(201)
	The AG/GG genotype of TLR4 rs4986790 is a protective factor against COVID-19 progression.	(202)
	Serum-soluble TLR4 is elevated in severe COVID-19 patients compared to non-severe patients.	(205)
	Up-regulated TLR4 in lethal COVID-19 patients can impair M2-like activities and lead to persistent inflammation and death.	(199)
	TLR4 is involved in the pathways of promoting platelet-related thrombosis in SARS-CoV-2.	(200)
	Elevated expression of TLR4 is observed in ICU COVID-19 patients compared to non-ICU patients.	(206)
	TLR4 Asp299Gly G allele may be related to more severe COVID-19 course and in-hospital death.	(203)
	There is no association between TLR4 polymorphisms and COVID-19 infections in the Kurdish Population.	(204)
	TLR4 Asp299Gly and Thr399lle minor alleles 299Gly(G) and 399lle(T) are related to COVID-19 severity, cytokine storm and mortality.	(238)
	TLR4-mediated NF-kB signaling pathway plays a role in upregulated inflammatory responses in COVID-19 patients.	(198)
	The response of TLR4 to SARS-CoV-2 may lead to a mass synthesis of pro-inflammatory mediators and contribute to hyperinflammation in severe COVID-19.	(190)
	TLR4 may contribute to immunosuppression following hyperinflammation in COVID-19 by reducing IL-8 secretion.	(191)
	Enhanced expression of TLR4 are associated with an unfavorable outcome in severe COVID-19 patients.	(195)
TLR7	Loss-of-function TLR7 mutations contribute to critical COVID-19 due to impaired RNA sensing ability and MyD88 signaling.	(18)
	There is no association between TLR7 polymorphism and SARS-CoV-2 infection in Korean females.	(211)
	Rare loss-of-function variants in TLR7 are associated with the downregulation of cytokine-mediated signaling in COVID-19 patients.	(208)
	The GG genotype of TLR7 rs3853839 may be a genetic risk factor for COVID-19 infection, which is highly severe and has poor clinical outcomes.	(210)
	TLR7 loss-of-function variants contribute to susceptibility in up to 2% of severe COVID-19 young males.	(207)
	TLR7 Gln11Leu SNP may contribute to SARS-CoV-2-induced hepatitis by impairing the initial immune response in a male child.	(209)
	TLR7 rs179008 polymorphisms may be risk factors for vulnerability to COVID-19.	(196)
	Hyperactivation of TLR7 may lead to higher severity of COVID-19.	(197)
	TLR7 rs179009 SNP is related to comorbidity among male COVID-19 patients.	(212)
TLR8	TLR8 rs5744080 and rs2159377 SNPs have no detrimental effect on COVID-19 symptoms.	(213)
	Hyperactivation of TLR8 may lead to higher severity of COVID-19.	(197)
	TLR8 Met1Val SNP contributes to COVID-19 severity in female COVID-19 patients.	(212)
TLR7/8	TLR7/8 may contribute to immunosuppression following hyperinflammation in COVID-19 by reducing IL-8 secretion.	(191)
TLR9	TLR9 rs5743836 variants are genetic risk factors for COVID-19 severity.	(189)
	TLR9 may contribute to immunosuppression following hyperinflammation in COVID-19 by reducing IL-8 secretion.	(191)
	Hyperactivation of TLR9 may lead to higher severity of COVID-19.	(197)

This table summarizes the involvement of various Toll-like receptors (TLRs) in COVID-19, highlighting their roles in disease progression, severity, and immune response. The table includes specific TLR types and their impact on COVID-19 pathology, emphasizing how TLRs contribute to recognizing SARS-CoV-2, mediating inflammatory responses, and influencing genetic susceptibility and disease outcomes. The table provides insights into potential therapeutic targets and genetic markers for understanding and managing COVID-19.

Sahanic et al. (190) further underscore TLR4’s importance by showing that SARS-CoV-2 activates the TLR4/MyD88 pathway in human macrophages, triggering strong pro-inflammatory responses linked to severe COVID-19. Their study suggests that TLR4 blockade can significantly reduce exaggerated inflammatory responses in macrophages infected with various SARS-CoV-2 variants, presenting a potential therapeutic target for severe COVID-19. Carreto-Binaghi et al. (191) find that IL-8 secretion via TLR2, TLR4, TLR7/8, and TLR9 receptors in blood cells from COVID-19 patients is significantly reduced at admission, indicating an impaired initial immune response. However, receptor functionality improves after 2 weeks of hospitalization, signaling recovery in the patient’s immune response. Additionally, compromised NOD2 receptor functionality at the onset of COVID-19 contributes to lower IL-8 levels, suggesting this impairment in innate immune receptors plays a role in the early-stage immunosuppression observed in severe COVID-19, which is later restored with continued hospitalization.

Several studies have collectively emphasized the significant role of TLR3 and related TLR polymorphisms in modulating the immune response to SARS-CoV-2, influencing COVID-19 severity and outcomes. Croci et al. (192) highlight the L412F polymorphism in TLR3 as a significant indicator of severe COVID-19, particularly in males. This genetic variant disrupts autophagy, impairing the innate immune response and exacerbating disease severity. Patients with this polymorphism exhibit reduced TNF production and a higher incidence of autoimmune conditions, underscoring the critical role of autophagy and immune regulation in the progression of severe COVID-19. Supporting these findings, Lieberum et al. (193) report that systemic TLR3 expression in blood negatively correlates with impaired lung function and neurological outcomes in severe COVID-19. Higher TLR3 levels are associated with less severe disease progression and better recovery. This study identifies TLR3 expression in blood, rather than bronchoalveolar lavage (BAL), as a stronger predictor of disease severity and long-term outcomes, highlighting its crucial role in the systemic immune response during severe COVID-19. In line with this, Dhangadamajhi and Rout (194) demonstrate that the TLR3 functional variant rs3775291 significantly increases susceptibility to and mortality from COVID-19. This variant impairs TLR3 expression and its ability to recognize SARS-CoV-2 dsRNA, leading to inadequate immune responses and higher disease severity and death rates across diverse populations. Further elucidating TLR3’s role, Menezes et al. (195) discovered that lower TLR3 expression in peripheral blood is associated with unfavorable outcomes in severe COVID-19 patients. This reduced expression correlates with a decreased interferon-gamma response, indicating a compromised antiviral defense mechanism in critically ill patients. Conversely, enhanced TLR4 expression in severe COVID-19 patients may reflect a compensatory mechanism for the impaired TLR3 response but also contribute to the heightened inflammatory state characteristic of severe COVID-19.

Additionally, Alseoudy et al. (196) report that TLR3 and TLR7 polymorphisms are linked to increased prevalence of COVID-19 pneumonia. Specifically, the TLR3 rs3775290 and TLR7 rs179008 polymorphisms significantly increase the risk of severe COVID-19 symptoms, suggesting their potential role in disease progression. However, these polymorphisms did not significantly correlate with COVID-19 pneumonia outcomes. Other factors such as male sex, low SPO2 levels, high INR, high LDH, and lymphopenia were identified as independent predictors of mortality, highlighting the complex interplay of genetic and clinical factors in disease prognosis. Bagheri-Hosseinabadi et al. (197) find that mRNA expression levels of TLR3, TLR7, TLR8, and TLR9 are significantly upregulated in nasopharyngeal epithelial cells of COVID-19 patients compared to controls. This upregulation correlates with disease severity and clinical markers of inflammation, suggesting that these TLRs play a critical role in COVID-19 pathogenesis and could serve as potential biomarkers for predicting disease severity. Collectively, these studies underscore the importance of TLR3 and other TLR polymorphisms in SARS-CoV-2 recognition and the immune response, highlighting their potential as therapeutic targets.

Several studies have demonstrated that TLR4 plays a critical role in the immune response to SARS-CoV-2 infection, with significant implications for disease severity and potential therapeutic strategies. TLR4-mediated signaling molecules are significantly upregulated in the PBMCs of COVID-19 patients compared to healthy controls, correlating with the role of S100A9, a TLR4 ligand, as a potential biomarker for severe COVID-19 due to its inverse correlation with serum albumin levels (198). In damaged pneumocytes and lung macrophages of patients who succumbed to COVID-19, TLR4 expression is significantly increased, suggesting a pivotal role in the hyper-inflammatory response observed in lethal cases of the disease (199). This inflammatory environment leads to a pathological shift in macrophage populations, with an upregulation of pro-inflammatory TLR4(+) macrophages and a depletion of GAL-3(+) macrophages, crucial for resolving inflammation and promoting tissue repair.

Furthermore, TLR4 plays a crucial role in platelet activation and subsequent thrombus formation in SARS-CoV-2 infection. The interaction of the SARS-CoV-2 spike protein with TLR4 induces oxidative stress and platelet activation, pivotal in COVID-19-associated thrombosis. Inhibition of TLR4 or its oxidative stress pathways significantly reduces platelet activation and thrombus growth, suggesting promising therapeutic strategies (200). Additionally, TLR4 activation leads to increased production of pro-inflammatory cytokines such as IL-6 and IL-1β, contributing to the severity of COVID-19. Inhibition of TLR4 significantly reduces the expression of these cytokines, highlighting the therapeutic potential of targeting TLR4 to manage severe COVID-19 cases (201).

Genetic studies have revealed that specific TLR4 polymorphisms correlate with COVID-19 severity. The TLR4 rs4986790 AG/GG genotype acts as a protective factor against severe COVID-19 outcomes, significantly reducing the risk of hospitalization, intensive care, or death. Patients with this genotype exhibit lower inflammatory markers, such as IL-6 and procalcitonin, associated with a more favorable prognosis (202). In contrast, the TLR4 (Asp299Gly and Thr399Ile) polymorphisms are significantly associated with an increased risk of severe COVID-19 and elevated levels of IL-6, indicating a heightened inflammatory response and severe disease progression (203). However, in the Kurdish population, no significant association was found between these polymorphisms and COVID-19 infection, suggesting that these SNPs may not influence COVID-19 susceptibility in this cohort (204). TLR4 also emerges as a potential biomarker and therapeutic target for COVID-19. Serum levels of soluble TLR4 (sTLR4) and soluble CD14 (sCD14) are significantly elevated in patients with severe COVID-19 compared to non-severe cases, indicating that sTLR4 and sCD14 could serve as promising markers for assessing clinical severity and potential therapeutic targets for managing severe COVID-19 (205). Moreover, TLR4 mRNA expression is significantly elevated in ICU COVID-19 patients compared to non-ICU patients, correlating with disease severity. High levels of inflammatory chemokines, such as IFN-*β* and CXCL13, present in ICU patients suggest these molecules could serve as biomarkers for severe COVID-19 and potential targets for immunotherapeutic strategies (206).

Research on TLR7 reveals its significant role in influencing COVID-19 severity, particularly among males. Fallerini et al. (207) discovered that rare loss-of-function variants in TLR7 are significantly linked to severe COVID-19 in males. These genetic alterations hinder the TLR7 signaling pathway, leading to diminished expression of type I and II interferon-related genes, crucial for antiviral defenses. The study found that 2.1% of severely affected males carried these TLR7 variants, while none of the asymptomatic individuals did, highlighting TLR7’s role in susceptibility to severe COVID-19 among young male patients. Mantovani et al. (208) identified similar rare loss-of-function variants in X-chromosomal TLR7 in young men with severe COVID-19, associated with impaired TLR7 signaling and reduced type I and II interferon responses. This genetic impairment led to significant downregulation of cytokine-mediated signaling, contributing to severe disease progression. Functional characterization of newly identified TLR7 variants in severely affected male patients demonstrated decreased mRNA levels in IFNα, IFNγ, RSAD2, ACOD1, IFIT2, and CXCL10 genes, revealing profound impairment of the TLR7 pathway.

Pessoa et al. (209) reported the first known case of SARS-CoV-2-induced hepatitis in a male child with the TLR7 Gln11Leu rs179008 polymorphism, suggesting this genetic variation may impair the initial immune response against the virus. This indicates that polymorphisms in the TLR7 gene, such as Gln11Leu rs179008, can negatively impact the innate immune response, leading to higher susceptibility to severe outcomes in viral infections like COVID-19. Naushad et al. (18) demonstrated that men with loss-of-function mutations in TLR7 have a significantly increased risk of severe COVID-19 due to disrupted viral RNA sensing and impaired MyD88 signaling. These findings highlight TLR7’s critical role in the innate immune response to SARS-CoV-2 and suggest that TLR7 agonists could potentially restore antiviral responses in individuals with these genetic variants. Interestingly, specific hypofunctional and neutral variants of TLR7 still maintain their ability to form the TLR7-MyD88-TIRAP complex and respond to TLR7 agonists, indicating potential for targeted therapies using TLR7 agonists to enhance immune responses in COVID-19 patients with specific TLR7 mutations.

El-Hefnawy et al. (210) found that the GG genotype of the TLR7 SNP (rs3853839) is significantly more prevalent in COVID-19 patients, suggesting a genetic predisposition to severe disease outcomes. They also observed that TLR7 mRNA expression levels were substantially higher in COVID-19 patients, particularly those with the GG genotype, correlating with increased disease severity and poorer clinical outcomes. In contrast, Zayed et al. (211) found no significant differences in the genotype or allele frequencies of the TLR7 rs864058 polymorphism between female COVID-19 patients and healthy controls, indicating this specific genetic variation in TLR7 is not associated with COVID-19 susceptibility in the Korean female population. This research suggests that while TLR7 plays a critical role in the innate immune response to SARS-CoV-2, the low-frequency TLR7 rs864058 polymorphism does not contribute to variations in disease severity or susceptibility, emphasizing the need to explore other genetic factors and TLR variants that might influence COVID-19 outcomes.

Research on the genetic factors influencing COVID-19 severity has highlighted the roles of TLR7 and TLR8 single nucleotide polymorphisms (SNPs). Bagci et al. (212) identified that the TLR8 Met1Val SNP is significantly associated with increased COVID-19 severity in female patients, particularly those admitted to the ICU. In male patients, the TLR7 rs179009 SNP A allele was more prevalent among individuals with comorbidities, suggesting its role in exacerbating disease severity under these conditions. Conversely, Mahallawi and Suliman (213) found that specific SNPs in TLR8, namely rs5744080 and rs2159377, did not affect the severity of COVID-19 symptoms in the Saudi population. Their findings suggest that the innate immune response, once activated, does not depend on the affinity level of the TLR8 receptor for identifying SARS-CoV-2 glycoprotein structures. DNA sequencing revealed that TLR8 is highly conserved among the Saudi population, with an average sequence homology of 99.63%, indicating minimal variation. This conservation implies that TLR8 mutations do not significantly impact the receptor’s function or the severity of COVID-19 symptoms in this population.

The collective findings from these studies illustrate the complex role of TLRs in COVID-19. TLR4 is consistently implicated in severe inflammatory and thrombotic responses, while TLR3 and TLR7 genetic variants are linked to differential disease outcomes and immune responses. These insights highlight the potential for targeted therapies, such as TLR agonists and inhibitors, to modulate immune responses and improve clinical outcomes in COVID-19 patients. Understanding the genetic and molecular mechanisms underlying TLR-mediated immune responses can guide the development of personalized treatments and preventive strategies for COVID-19 and other viral infections.

Furthermore, all mentioned studies were conducted to elucidate the role of TLRs in the neuroinflammation and neuroinvasion associated with SARS-CoV-2; however, they lack conclusive experimental evidence in humans to substantiate these mechanisms. Addressing this limitation necessitates conducting human studies, such as postmortem analyses of brain tissues from COVID-19 patients, alongside advanced transcriptomic and proteomic investigations, to verify TLR-mediated pathways. Longitudinal cohort studies across diverse populations are imperative to validate the influence of TLR polymorphisms on disease severity and long-term neurological outcomes. While TLR activation is implicated in the disruption of the BBB, additional studies utilizing *ex vivo* BBB models or imaging techniques are required to elucidate these interactions in humans. Although TLR-targeted therapies exhibit promise in preclinical investigations, robust clinical trials are essential to assess their safety and efficacy in alleviating neurological complications. Moreover, there is a pressing need for research to delineate specific cytokine profiles associated with TLR activation in neuroinflammation, correlating these profiles with cerebrospinal fluid and serum markers in affected patients. The interplay between TLRs and other innate immune receptors, such as NOD-like receptors, remains another underexplored domain, potentially uncovering synergistic mechanisms in SARS-CoV-2-induced inflammation. Limited data exist regarding the long-term cognitive and neurological ramifications of TLR-mediated neuroinflammation, thus necessitating neuropsychological assessments and imaging within long-COVID cohorts. Besides, the molecular underpinnings of sex-specific differences in TLR-related immune responses warrant further examination. Addressing these research gaps through multidisciplinary approaches will enhance the scientific framework for comprehending TLR-mediated mechanisms in SARS-CoV-2 infection and will inform the development of personalized therapeutic and preventive strategies.

## 6 Toll-like receptors in neurodegenerative outcomes of long COVID

Considering the putative shared mechanisms by which TLRs contribute to the pathogenesis of neurodegenerative diseases and the host immune response to SARS-CoV-2 infection suggest potential synergistic pathways that may underlie the development of neurodegenerative phenotypes observed in a subset of Long COVID patients. Persistent neuroinflammation is a hallmark of ‘Long COVID’ (214), and TLRs are crucial in mediating this inflammatory response (67). Activation of TLRs, particularly TLR2 and TLR4, by SARS-CoV-2 infection or the resulting inflammatory mediators, can lead to sustained microglial activation and the release of pro-inflammatory cytokines in the CNS (183). This chronic neuroinflammation can contribute to neuronal dysfunction and degeneration. Studies have documented that amyloid precursor protein (APP) facilitates SARS-CoV-2 virus entry into cells and enhances Aβ-associated pathology (215). In some cases of Long COVID, the potential for SARS-CoV-2 infection to induce the aggregation of certain proteins, such as Aβ, has been observed (216–219). These protein aggregates can act as endogenous ligands for TLRs, further activating inflammatory pathways and exacerbating neurodegeneration (220). The interplay between protein aggregation and TLR-mediated signaling may play a role in developing neurodegenerative phenotypes in Long COVID (221). Further, TLR activation has been linked to synaptic dysfunction and impairment of synaptic plasticity (222), which are early events in the pathogenesis of neurodegenerative diseases. The dysregulation of TLR signaling observed in Long COVID may contribute to the loss of synaptic connections and the impairment of neuronal communication, leading to cognitive impairment and other neurological manifestations.

However, despite the importance of TLRs, there is a significant dearth of comprehensive studies on the role of TLRs in post-COVID-19 conditions, underscoring the need for further investigation in this area. A study by Noor Eddin et al. identified a novel hemizygous loss-of-function variant in the TLR7 gene in a pediatric patient who experienced severe neurological deterioration following COVID-19 infection. This finding suggests a critical role for TLR7 in mediating immune response and neurological outcomes post-infection, emphasizing the importance of genetic screening for TLR7 variants in predicting COVID-19 severity and long-term effects (223). Complementing this, a study by Fontes-Dantas et al. (16) highlighted the role of TLR4 in mediating long-term cognitive dysfunction, a significant symptom of post-COVID-19 syndrome. The study demonstrated that infusion of the SARS-CoV-2 Spike protein into the brains of mice induced late cognitive dysfunctions, marked by neuroinflammation and synapse loss. This cognitive impairment was mediated via TLR4 signaling, with genetic or pharmacological blockage of TLR4 protecting against synapse elimination and memory dysfunction. Moreover, the study identified that in a cohort of COVID-19 patients, those with the TLR4-2604G > A GG genotype (rs10759931) were associated with poorer cognitive outcomes, underscoring TLR4’s pivotal role in long-term neurological effects post-COVID-19.

Collectively, these studies underscore the significant role of TLRs, especially TLR4 and TLR7, in the pathogenesis of post-COVID-19 conditions. They highlight the potential mechanisms these receptors contribute to prolonged inflammation and tissue damage, leading to the diverse and persistent symptoms observed in ‘Long COVID’. The findings suggest that targeting these receptors could offer therapeutic potential in mitigating the long-term effects of COVID-19. However, the current body of research remains limited, necessitating further studies to comprehensively understand the molecular underpinnings and develop targeted treatments for post-COVID-19 syndrome.

## 7 Potential to repurpose the regimens targeting TLRs for treating post-COVID-19 syndrome

The aftermath of COVID-19, often termed post-COVID-19 syndrome or Long COVID, has prompted a deeper exploration into novel therapeutic strategies. Targeting Toll-like receptors (TLRs) and their signaling pathways emerges as a promising approach due to the crucial role these receptors play in the innate immune response and inflammation.

Several studies emphasize the significance of TLR4 in COVID-19 pathogenesis. TLR4 recognizes viral components, triggering a cascade that results in the production of pro-inflammatory cytokines such as IL-6 and TNF-*α*, which are pivotal in the cytokine storm observed in severe COVID-19 cases (224). Thus, therapeutic agents that inhibit these TLR signaling might potentially reduce chronic inflammation and modulate immune responses, thereby preventing tissue damage and promoting recovery. Another promising strategy involves using nucleic acid-based therapeutics, such as small interfering RNAs (siRNAs) and antisense oligonucleotides (ASOs), which can specifically target and downregulate the expression of TLRs or their signaling components. These therapies might offer a high degree of specificity and can be designed to silence genes involved in aberrant TLR signaling, thereby mitigating the detrimental effects of chronic inflammation. One study discusses using BZL-sRNA-20, an oligonucleotide targeting TLR4, which has shown efficacy in reducing acute lung injury (ALI) and acute respiratory distress syndrome (ARDS) in mice models. This suggests that targeting TLR4 could potentially mitigate severe respiratory complications associated with COVID-19 (225).

Furthermore, modulation of TLR signaling using synthetic ligands or agonists may help recalibrate the immune response, promoting a more balanced and controlled inflammatory environment. Agonists of these receptors can enhance antiviral responses, whereas antagonists might be used to suppress excessive inflammation. For instance, the antagonist M5049 (enpatoran), which targets TLR7 and TLR8, is currently under clinical evaluation and has shown promise in reducing inflammatory responses in COVID-19 patients (226, 227). A study by Proud et al. investigated the prophylactic use of the TLR2/6 agonist INNA-051 in a ferret model to reduce viral shedding of SARS-CoV-2. Prophylactic intranasal administration of INNA-051 markedly decreased viral RNA levels in the nose and throat of infected ferrets by up to 96%, highlighting its potential to limit person-to-person transmission of COVID-19. These findings strongly support the further development of TLR2/6 agonists as a therapeutic approach to activate innate immune responses at mucosal surfaces, thereby providing a rapid and effective defense against SARS-CoV-2 (228). The conjugation of the TLR1/2 agonist Pam3CSK4 to the SARS-CoV-2 receptor-binding domain (RBD) significantly boosted both the antibody and cellular immune responses against RBD. This approach effectively inhibited RBD-ACE2 binding and provided protection against SARS-CoV-2 and its variants, underscoring the potential of TLR1/2 agonists in enhancing vaccine efficacy (229). PUL-042, a combination therapeutic currently undergoing clinical trials, comprises the TLR2/6 ligand Pam2CSK4 and the TLR9 ligand ODN M362. It is being evaluated for its efficacy as a prophylactic agent in reducing the infection rate and progression of COVID-19 (230, 231). Various TLR modulators are undergoing clinical trials to assess their efficacy in treating COVID-19. For instance, TAK-242 (resatorvid), a TLR4 antagonist, has shown the potential to reduce the inflammatory response in preclinical models (190, 232). Further, in a study investigating the link between TLR7 loss-of-function (LOF) mutations and the severity of COVID-19, researchers discovered that TLR7 agonists can effectively bind to hypofunctional and neutral TLR7 variants. These agonists stimulate TLR7-MyD88-TIRAP-mediated signaling, thereby restoring antiviral responses. Agonists capable of binding to most TLR7 variants have the potential to serve as effective adjuvants in vaccines (18).

Similarly, other TLR-targeted therapies, including agonists and antagonists, are being explored for their ability to modulate the immune response and mitigate the severity of COVID-19. A recent study has also highlighted the development of inulin acetate-based nanoparticles (InAc-NPs) as a TLR4 agonist for intranasal vaccination, which significantly enhances both systemic and mucosal immune responses, indicated by elevated IgG1, IgG2a, and sIgA levels. InAc-NPs effectively activate TLR4 on macrophages, leading to a robust immune response and demonstrating their potential as a novel adjuvant for mucosal vaccines (233). Likewise, recent research successfully developed a SARS-CoV-2 spike protein subunit vaccine incorporating a dual TLR ligand liposome adjuvant, which demonstrated high efficacy in mice by inducing potent systemic neutralizing antibodies and significant levels of anti-spike IgA. The vaccine’s efficacy was further evidenced by the complete protection of mice from a lethal viral challenge, highlighting its potential for robust protective immunity against COVID-19 (234). A recent study found that stimulating PBMCs from moderate COVID-19 patients with a TLR8 agonist and the spike protein (SP) of SARS-CoV-2 significantly increased the frequency of CD3 + IFN-*β* + T cells and upregulated IFN-β gene expression compared to healthy controls. Notably, the TLR8 agonist induced the highest frequency of IFN-β-producing T cells in moderate patients, highlighting its potential to enhance antiviral immune responses in COVID-19 (235).

Targeting TLRs offers a dual benefit: enhancing antiviral responses during the acute phase of infection and mitigating chronic inflammation associated with post-COVID-19 syndrome. Modulating TLR pathways could address the prolonged immune activation and inflammation seen in long COVID-19, thereby reducing symptoms and improving patient outcomes. The strategic targeting of TLRs and their signaling pathways presents a viable therapeutic avenue for managing both acute and long-term complications of COVID-19. By fine-tuning the immune response, it is possible to enhance antiviral defenses while mitigating the harmful effects of hyperinflammation. As research progresses, TLR-targeted therapies could become integral components of the therapeutic arsenal against COVID-19 and its lingering effects. No regulatory TLR drugs have been approved for clinical use due to insufficient studies on their efficacy and safety and the lack of standard control treatments for COVID-19 (236). Additionally, the variability in COVID-19 severity among patients complicates the understanding of the disease.

Despite the promising potential of therapies targeting TLRs in addressing COVID-19 and its associated complications, it is imperative to recognize several limitations and potential adverse effects. A predominant concern pertains to the risk of off-target effects, wherein the therapeutic modulation of TLR signaling may unintentionally exacerbate systemic inflammation. For example, excessive activation of TLRs by agonists could enhance the production of pro-inflammatory cytokines, potentially resulting in cytokine storm-like conditions in susceptible patients. Conversely, the inhibition of TLRs may diminish critical antiviral defenses, thereby increasing the risk of secondary infections. Furthermore, these interventions might disrupt the delicate equilibrium between immune activation and regulation, culminating in immune dysregulation and possible autoimmune reactions. Prolonged suppression of TLR pathways could impair the immune system’s ability to resolve infections or inflammation, while overstimulation may contribute to persistent inflammatory signaling and autoimmune pathologies. Another substantial challenge resides in the variability of patient responses, influenced by factors such as genetic polymorphisms, comorbidities, and the severity of the disease. This variability complicates the formulation of standardized therapeutic approaches and underscores the necessity for personalized strategies. Finally, the absence of extensive clinical trials and regulatory approvals for TLR-targeted pharmaceuticals in COVID-19 treatment emphasizes the urgent need for further research to ascertain their safety, efficacy, and long-term effects in the management of this disease and its prolonged sequelae.

## 8 Conclusion and future directions

The COVID-19 pandemic has led to significant public health concerns regarding the long-term neurological impacts of the disease. This review examines the role of TLRs in mediating the immune response to SARS-CoV-2. The possible intricate interactions between TLRs and post-COVID-19 neurodegenerative processes highlight the crucial role these receptors play in long-term neurological consequences. The evidence demonstrates that TLRs are essential mediators of the immune response to SARS-CoV-2, contributing to both the immediate and long-term effects of infection. Specifically, certain TLRs have been identified as critical players in the inflammatory response, potentially exacerbating neuroinflammation and driving the development of neurodegenerative diseases, such as AD and PD, in individuals who have recovered from acute COVID-19. Furthermore, genetic polymorphisms in TLRs have been linked to variations in COVID-19 severity and the persistence of neurological symptoms. These findings suggest that individuals with specific genetic profiles may be more susceptible to severe outcomes and long-term complications, highlighting the need for personalized approaches in managing post-COVID-19 conditions.

Given the pivotal role of TLRs in post-COVID-19 neurodegenerative disorders, future research should focus on several key areas to improve understanding and therapeutic strategies. Developing specific inhibitors or modulators of TLR signaling pathways offers a promising therapeutic avenue, with potential drugs that selectively inhibit TLR activation from reducing chronic inflammation and preventing neurodegenerative disease progression in post-COVID-19 patients. Genetic screening for TLR polymorphisms could inform personalized medicine approaches, tailoring interventions based on individual risk profiles. Longitudinal studies are essential to understand the temporal relationship between TLR activation, neuroinflammation, and neurodegenerative symptom onset. Further mechanistic studies using advanced imaging, animal models, and *in vitro* systems are needed to elucidate how TLR signaling contributes to CNS pathology. Additionally, exploring immunomodulatory therapies that balance protective and pathological aspects of TLR activation could offer dual benefits in managing post-COVID-19 neurological outcomes. Integrating these findings into public health strategies is crucial for monitoring and managing neurological health in COVID-19 survivors, thereby addressing the long-term impact of the pandemic.
